# Biochemical characterization of the human ubiquitous glucose‐6‐phosphatase in neutrophil granulocytes

**DOI:** 10.1002/2211-5463.13924

**Published:** 2024-11-15

**Authors:** Zsigmond Lédeczi, Klaudia Németh, Tamás Kardon

**Affiliations:** ^1^ Department of Molecular Biology Institute of Biochemistry and Molecular Biology, Semmelweis University Budapest Hungary

**Keywords:** endoplasmic reticulum, enzyme kinetics, glucose‐6‐phosphatase catalytic subunit 3, glucose‐6‐phosphate transporter, neutrophil granulocytes, substrate specificity

## Abstract

Glucose‐6‐phosphatase‐β (G6PC3) is a ubiquitous phosphatase present in the endoplasmic reticulum, which, unlike G6PC1, is not responsible for maintaining blood glucose level under starvation. Recently, G6PC3 has been shown to play an important role in neutrophil granulocytes, eliminating the toxic metabolite 1,5‐anhydroglucitol‐6‐phosphate. The present study aimed to look for alternative substrates for the enzyme and outline the expression changes in the parts of this multicomponent system during neutrophil granulocyte differentiation. We determined the kinetic characteristics of recombinant human G6PC3 towards different sugar phosphates, and the transport of these compounds was also measured in rat liver microsomes. We found that all investigated sugar phosphates are substrates for G6PC3, although their microsomal transport is much slower than that of glucose‐6‐phosphate. Using the HL‐60 promyelocytic leukemia cell line as an *in vitro* model system for myeloid differentiation, we found no significant differences in enzyme expression and phosphatase activity latency between undifferentiated and differentiated cells. Our results provide novel insights into the possible role of G6PC3 in the dephosphorylation of alternative sugar phosphates or their metabolites synthesized in the endoplasmic reticulum and confirm the potential feature of the enzyme in the promyelocytic stage as well. These findings contribute to our knowledge of intracellular carbohydrate metabolism of neutrophil granulocytes, which facilitates further research directions to better understand the underlying mechanisms of neutropenias.

## Graphical abstract

Glucose‐6‐phosphatase‐β (G6PC3) is a ubiquitous phosphatase located in the endoplasmic reticulum, which plays a crucial role in neutrophil granulocytes by eliminating 1,5‐anhydroglucitol‐6‐phosphate. However, the enzyme appears to be a non‐specific phosphohydrolase with constant expression throughout neutrophil differentiation. Understanding the role of G6PC3 in the dephosphorylation of alternative sugar phosphates may contribute to a deeper understanding of intracellular carbohydrate metabolism.

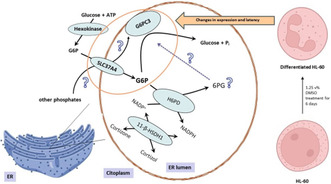

**Abbreviations**
1,5‐AHG: 1,5‐anhydroglucitol
1,5‐AHG6P: 1,5‐anhydroglucitol‐6‐phosphate
6PG: 6‐phosphogluconate
ER: endoplasmic reticulum
F6P: fructose‐6‐phosphate
G6P: glucose‐6‐phosphate
G6PC3: glucose‐6‐phosphatase‐β
G6PT/SLC37A4: glucose‐6‐phosphate transporter
GSD1b: glycogen storage disease type 1b
M6P: mannose‐6‐phosphate
R5P: ribose‐5‐phosphate
S6P: sorbitol‐6‐phosphate
SCN4: severe congenital neutropenia type 4

In humans, three glucose‐6‐phosphatase isoforms have been identified, encoded by the G6PC gene‐family, consisting of G6PC1, G6PC2 and G6PC3 genes. They are all transmembrane enzymes localized in the endoplasmic reticulum (ER) with their catalytic site facing the lumen. They work in tandem with the glucose‐6‐phosphate transporter (G6PT/SLC37A4), which facilitates the entry of cytosolic substrates. The three phosphatase enzymes, however, differ in their tissue distribution and substrate affinity [1]. G6PC1, the classic glucose‐6‐phosphatase, mainly present in the liver and the kidneys, is responsible for maintaining blood glucose concentration under starvation [2]. G6PC2 is expressed by pancreatic islet β cells and plays a role in the regulation of insulin secretion [3, 4]. G6PC3 is a ubiquitous enzyme, which is not involved in the blood glucose homeostasis [4, 5, 6, 7, 8].

G6PC3 is mostly expressed in the brain, kidneys, heart and skeletal muscle [5]. The exact function of this G6Pase isoform in each tissue has not been explored fully yet. It has recently been shown to play a pivotal role in neutrophil granulocytes by eliminating the toxic metabolite 1,5‐anhydroglucitol‐6‐phosphate (1,5‐AHG6P). This substance is generated from the food derived polyol 1,5‐anhydroglucitol (1,5‐AHG) through phosphorylation, mainly by hexokinase isoforms and ADP‐dependent glucokinase. Under physiological circumstances, 1,5‐AHG6P is transported into the endoplasmic reticulum by G6PT, where it goes through dephosphorylation by G6PC3. Dysfunctions in the transporter‐phosphatase system cause 1,5‐AHG6P accumulation in the cytosol, which competes with glucose‐6‐phosphate (G6P) in multiple enzymatic processes, leading to the inhibition of glycolysis and the pentose phosphate pathway. Because the energy metabolism of neutrophil granulocytes mostly relies on glycolysis, these cells exhibit increased apoptosis and disturbed maturation [9]. The lack of G6PT activity is the underlying cause of neutropenia in glycogen storage disease type 1b (GSD1b) [10, 11], whereas the missing G6PC3 activity is responsible for severe congenital neutropenia type 4 (SCN4) [12, 13, 14]. Although the most life‐threatening consequence of SCN4 is neutropenia, leading to recurring bacterial infections, the disease has a broad phenotypic spectrum. Patients may also exhibit intermittent thrombocytopenia, minor facial dysmorphism, a prominent superficial venous pattern and a high incidence of congenital cardiac and uro‐genital malformations among other rare symptoms [14]. Nevertheless, in both SCN4 and GSD1b, neutropenia can be alleviated by oral SGLT‐2 inhibitor therapy, which inhibits the renal reabsorption of 1,5‐AHG, effectively decreasing intracellular 1,5‐AHG6P concentrations [15, 16, 17, 18, 19].

Besides its expanding role in neutrophil granulocytes as a metabolic repair enzyme, G6PC3 has been recently proposed to be linked to the progression and metabolic reprogramming of certain cancers. In glioblastoma, an aggressive and highly heterogeneous primary brain tumor characterized by intratumoral hypoxia, high G6PC3 expression was linked to poor overall patient survival [20, 21]. By altering the self‐renewal capacity of tumor cells and potentially affecting chemotherapeutic responses, the G6PC3 phosphatase system and alternative substrate research may offer promising avenues for developing novel treatment strategies for this cancer type.

Regarding the substrate affinity of G6PC3, the first attempts to show the G6Pase activity in transiently transfected COS7 cells did not succeed [5, 7]; however, G6PC3 exhibits G6Pase activity when expressed at higher levels. In comparison with the G6PC1 activity in rat liver, the optimal pH for G6PC3 is lower, the *K*_m_ is higher (~2 mm at pH 6.5) and the *V*_max_ is 7‐fold lower [4]. Recently, it has been shown that G6PC3 can hydrolyze a variety of phosphorylated carbohydrates. The study focused on the role of 1,5‐AHG6P, but ribose‐5‐phosphate proved to be a good substrate as well. Although the role of the G6PC3‐G6PT system in eliminating 1,5‐AHG6P is well‐established [9], little is known about the significance of other potential substrates.

In the present study, we aimed to kinetically characterize G6PC3 for alternative substrates using overexpressed human recombinant protein in HEK293T cells and measure their ER intraluminal transport on rat liver microsomes. We had hypothesized that G6PC3 has a wider substrate specificity than previously uncovered. Furthermore, in the present study, we used the human myeloid HL‐60 cell model to investigate how the differentiation of the promyelocytes into mature neutrophil granulocytes affects the protein expression and phosphatase activity of the transporter/phosphatase system. The results might support our hypothesis that a constant G6PC3 phosphatase activity in myeloid progenitor cells has as much importance as in peripheral neutrophil granulocytes.

## Materials and methods

### Cell culture and differentiation

Human embryonic kidney cells (HEK293T) (CRL‐3216; ATCC, Manassas, VA, USA) were routinely maintained in Dulbecco's modified Eagle's medium high glucose with l‐glutamine, without sodium‐pyruvate (Cat. No. 41965‐039; Gibco, Waltham, MA, USA), supplemented with 10 vol% fetal bovine serum (Cat. No. 10500‐064, Gibco), 100 U·mL^−1^ penicillin, 100 μg·mL^−1^ streptomycin and 0.25 μg·mL^−1^ amphotericin‐B (Cat. No. 15240‐096; Gibco) at 37 °C with 5% CO_2_.

The HL‐60 human acute promyelocytic leukemia cell line (Cat. No. 98070106; Sigma‐Aldrich, St Louis, MO, USA) was cultured in RPMI 1640 with l‐glutamine (Cat. No. 21875‐034; Gibco) supplemented with 10% fetal bovine serum (Cat. No.10500‐064; Gibco), 100 U·mL^−1^ penicillin, 100 μg·mL^−1^ streptomycin and 0.25 μg·mL^−1^ amphotericin‐B (Cat. No. 15240‐096; Gibco) at 37 °C with 5% CO_2_. For differentiation, 2.5 × 10^5^·mL^–1^ HL‐60 cells were seeded and incubated for 6 days in the RPMI 1640 medium mentioned above, additionally supplemented with 1.25% dimethylsulfoxide (Cat. No. D2650; Sigma‐Aldrich). On the third day, the medium was changed.

### Mammalian expression vector

G6PC3 cDNA was subcloned into the *Xho*I/*Eco*RI sites of a pEGFP‐N1 vector (Cat. No. 6085‐1; Takara Bio, Shiga, Japan). G6PC3 cDNA was amplified using universal human cDNA template and PCR primers with specific sites for restriction enzymes added to the 5′ end (*Xho*I and *Eco*RI) (forward primer: AAGCATCTCGAGATGGAGTCCACGCTGGGC; reverse primer: ATCCGGAATTCCGGAAGAGTGGATGGGCGG). Following the PCR amplification, the products were purified using the PureLink PCR purification kit (Cat. No. K220001; Invitrogen, Waltham, MA, USA). Purified PCR products were digested by *Xho*I (Cat. No. ER0692; Thermo Fisher Scientific, Walthem, MA, USA) and *Eco*RI (Cat. No. ER0271; Thermo Fisher Scientific) and then phosphorylated by T4 PNK (Cat. No. M0201S; New England Biolabs, Ipswich, MA, USA). The pEGFP‐N1 vector was cut with the same restriction enzymes and purified using the PureLink PCR purification kit. Finally, ligation was performed using T4 DNA ligase (Cat. No. M0202S; New England Biolabs) with overnight incubation at 16 °C, in accordance with the manufacturer's instructions.

Transformation was carried out using DH5α‐T1 competent cells (Cat. No. 12297‐016; Invitrogen), in accordance with the manufacturer's instructions. Transformed cells were plated on premixed lysogeny broth with agar (Cat. No. L2897; Sigma‐Aldrich) supplemented with 50 μg·mL^−1^ kanamycin (Cat. No. 15160054; Invitrogen) and incubated at 37 °C overnight. The next day, 250 mL lysogeny broth media [1% tryptone (Cat. No. T7293; Sigma‐Aldrich), 0.5% yeast extract (Cat. No. Y1625; Sigma‐Aldrich), 1% NaCl, 50 μg·mL^−1^ kanamycin (Cat. No. 15160054; Invitrogen)] was inoculated with a single colony and incubated overnight on a shaker at 37 °C. Plasmid purification was performed using GeneJET Plasmid Maxiprep Kit (Cat. No. K0491; Thermo Fisher Scientific). *D*_260/280_ of the purified plasmid DNA was measured using a Nanodrop spectrophotometer (NanoDrop One; Thermo Fisher Scientific). The construct sequence was verified by Sanger sequencing.

### Transient transfection

HEK293 cells were seeded in 25 cm^2^ cell culture flasks (Cat. No. 169900; Thermo Fischer Scientific) and transfected at 60–70% confluency with pEGFP‐N1 expression plasmids containing the coding sequence of G6PC3, using jetPRIME Transfection Reagent Kit (Cat. No. 114‐75C; Polyplus‐Transfection, Illkirch, France), in accordance with the manufacturer's instructions.

### Phosphatase activity assay

H293T and HL60 cells grown in 75 cm^2^ flasks (Cat. No. 156800; Thermo Fischer Scientific) were harvested and centrifuged at 300 ***g*** for 5 min. Supernatant was thoroughly drawn off, and the pellet was resuspended in 400 μL of homogenization buffer, containing 25 mm Hepes pH 7.2, 25 mm KCl, 300 mm saccharos, and a 10 vol% protease inhibitor cocktail (Cat. No. P8465; Sigma‐Aldrich). Suspensions were sonicated on ice five times for 10 s (Sonic 300 dismembrator; Artek Systems, Helsinki, Finland). Cell lysates were stored in 400 μL aliquots at −80 °C until they were used for enzymatic measurements. Each sample was thawed once before use. Protein concentration of the samples was measured using Pierce™ BCA Protein Assay Kit (Cat. No. 23225; Thermo Fischer Scientific). Phosphatase activity assays were carried out in a buffer containing 50 mm Mops, pH 6.5; 25 mm CaCl_2_; 0.5 mm MnCl_2_; 20 mm KCl; 20 mm NaCl; 2.5 mm EDTA, and the required substrate concentration. Investigated substrates were glucose‐6‐phosphate (G6P) (Cat. No. G6526; Sigma‐Aldrich), 6‐phospho‐gluconate (6PG) (Cat. No. P7877; Sigma‐Aldrich), fructose‐6‐phosphate (F6P) (Cat. No. F3637; Sigma‐Aldrich), ribose‐5‐phosphate (R5P, Cat. No. R7750; Sigma‐Aldrich), mannose‐6‐phosphate (M6P, Cat. No. M3655; Sigma‐Aldrich) and sorbitol‐6‐phosphate (S6P) (Cat. No. S1753; Sigma‐Aldrich). Alamethicin (Cat. No. A4665; Sigma‐Aldrich) was also added to the buffer to permeabilize membranes (0.1 mg·mg^–1^ protein). The reaction started with the addition of the sample (final protein concentration: 0.3 mg·mL^−1^). The reaction environment was incubated at 37 °C for 5–20 min, depending on the substrate. The reaction was stopped by adding the inorganic phosphate (Pi) reaction mixture, for which the initial and final concentrations were measured using the sensitive assay of Morrison [22] with a slight modification. The reagent mixture, which was prepared daily, contained one part of 10% ascorbic acid (Cat. No. A92902; Sigma‐Aldrich), two parts of 10 vol% sodium‐dodecyl‐sulfate (Cat. No. 161‐0418; Bio‐Rad, Hercules, CA, USA) and six parts of 0.42% ammonium molybdate (Cat. No. 0242; Reanal, Budapest, Hungary) in 1 n sulfuric acid. A total of 900 μL of reagent was added to 100 μL sample and, after 20 min incubation at 46 °C, the optical density was measured at 820 nm wavelength against a blank containing 900 μL of reagent and 100 μL of buffer. Phosphatase activities for the different substrates (G6P, R5P, 6PG, F6P, S6P and M6P) were calculated as the amount of inorganic phosphate produced in 1 min by 1 mg of cell lysate protein.

For a negative control, we used H293T cells transfected with the empty pEGFP‐N1 vector. To correct the values for the non‐specific phosphatase activity, the GFP overexpressed sample activity values were subtracted from the GFP‐G6PC3 overexpressed sample activities. The *K*_m_ and *V*_max_ were determined by non‐linear regression analysis. Phosphatase activity of microsomes was also measured with the method mentioned above. The final protein concentration was 0.5 mg·mL^−1^. The phosphatase activity of intact and alamethicin (0.1 mg·mg^–1^ protein) permeabilized microsomes were used to calculate activity latency in control and differentiated HL‐60 cells. Latency was calculated as: (Activity_permeabilized_ − Activity_intact_)/Activity_intact_ × 100.

### Microsomal fraction isolation

Rat liver microsomes were prepared from 24 h fasted male Sprague–Dawley rats (180–230 g) using differential centrifugation as reported by Henne and Söling [23]. For HL‐60 derived microsomes, cells were harvested and washed in ice cold phosphate‐buffered saline. Cells were resuspended in 1 mL of lysis buffer (0.3 m sucrose, 20 mm Hepes‐KOH pH 7.2) followed by sonication on ice five times for ten s (Sonic 300 dismembrator; Artek Systems). After centrifugation at 1000 ***g*** for 10 min, the supernatant was spun for 10 min at 12 000 ***g***. The 12 000 ***g*** supernatant was spun for 60 min at 100 000 ***g***. Microsomal fractions were resuspended in a buffer containing 100 mm KCl, 20 mm NaCl, 1 mm MgCl_2_, 20 mm MOPS, pH 7.2, and 10 vol% protease inhibitor cocktail (Cat. No. P8465; Sigma‐Aldrich). The suspensions were rapidly frozen and maintained under liquid nitrogen until used. Protein concentration of microsomes was measured using Pierce™ BCA Protein Assay Kit (Cat. No. 23225; Thermo Fisher Scientific).

### Transport measurement

The permeability of rat liver microsomes was investigated by a light scattering technique described by Marcolongo *et al*. [24]. Microsomal vesicles were incubated for 2 h in a hypotonic medium (5 mm K‐Pipes, pH 7) at a final protein concentration of 30–35 μg·mL^−1^. Microsomal intactness was verified using sucrose, which is a known poor permeant. The measurement started by adding 50 μL of 1 m solutions of the compounds investigated to 1.5 mL of the incubation volume. At the end of the measurement, microsomes were permeabilized with 20 μg of alamethicin. The osmotically‐induced changes in light scattering were measured at 400 nm at a right angle to the incoming light beam using a fluorimeter (Cary Eclipse Fluorescence Spectrophotometer; Agilent, Santa Clara, CA, USA) equipped with a recorder, a temperature‐controlled cuvette holder (37 °C) and a magnetic stirrer.

### Immunoblot analysis

HL‐60 cells were harvested and washed in phosphate‐buffered saline. Cells were resuspended in 100–200 μL of homogenization buffer followed by sonication on ice for 10 s five times (Sonic 300 dismembrator; Artek Systems). Equal amount of protein per sample (25 μg) was subjected to heat treatment at 95 °C for 5 min in loading dye (25 mm Tris‐Cl, pH 7.5; 50 vol% glycerol; 10% SDS; 5% bromophenol‐blue) followed by PAGE (10 vol%) in Laemmli buffer (Cat. No. P170065; Serva, Heidelberg, Germany). After electrophoresis, samples were electro transferred onto poly(vinylidene difluoride) membranes (Cat. No. IPV00010; Immobilon, Darmstadt, Germany). Membranes were blocked in 8 mL of Tris buffered saline, pH 7.6, with 0.1 vol% Tween (TBS‐T) containing 5% non‐fat milk powder at room temperature for 60 min. Incubation was performed with anti‐CD11b 1:1000 (Cat. No. PA5‐79533; Invitrogen), anti‐G6PC3 1:1000 (Cat. No. ab‐133 964; Abcam, Cambridge, UK), anti‐SLC37A4 1:1000 (Cat. No. ab‐80 463; Abcam) and anti‐GAPDH 1:40000 (Cat. No. sc‐32 233; Santa Cruz Biotechnology, Santa Cruz, CA, USA) primary antibodies overnight at 4 °C. Thereafter the membranes were washed in 0.5% TBS‐Tween buffer three times for 10 min, followed by incubation with the appropriate horseradish peroxidase‐linked anti‐rabbit 1:5000 (Cat. No. 7074S; Cell Signaling, Danvers, MA, USA) or anti‐mouse 1:5000 (Cat. No. 7076S; Cell Signaling) secondary antibodies for 90 min at room temperature. After washing for 3 × 10 min, membranes were developed using SuperSignal West Pico Chemiluminescent Substrate (Cat. No. 34578; Thermo Fisher Scientific). Densitometric analysis was carried out using a GelDoc™ reader and ImageLab™ (Bio‐Rad) software.

### Statistical analysis

The statistical and enzyme kinetic analyses were performed in Prism, version 10 (GraphPad Software Inc., San Diego, CA, USA). For the Michaelis–Menten enzyme kinetic analysis of recombinant G6PC3, we plotted substrate saturation curves with non‐linear regression, where each point represents the mean ± SEM of a triplicate measurement. The goodness of fit was determined with the standard deviation of the residuals (Sy.x). *K*_m_ and *V*_max_ values (± SE) for each substance were determined using non‐linear regression analysis. Microsomal phosphatase activity assays to determine latency were carried out in three independent measurements, each in a triplicate. Latencies were plotted as the mean ± SEM, whereas the mean ± SD and the confidence intervals are reported in the Supporting information (Table S1). Densitometric analysis of Western blots was made based on three independent experiments; relative values were plotted as the mean ± SEM. To check whether the differences in latency and protein expression between control and differentiated cells are significant, an unpaired Student's *t*‐test was performed. *P* < 0.05 was considered statistically significant.

## Results

### Enzyme kinetics

**Fig. 1:**
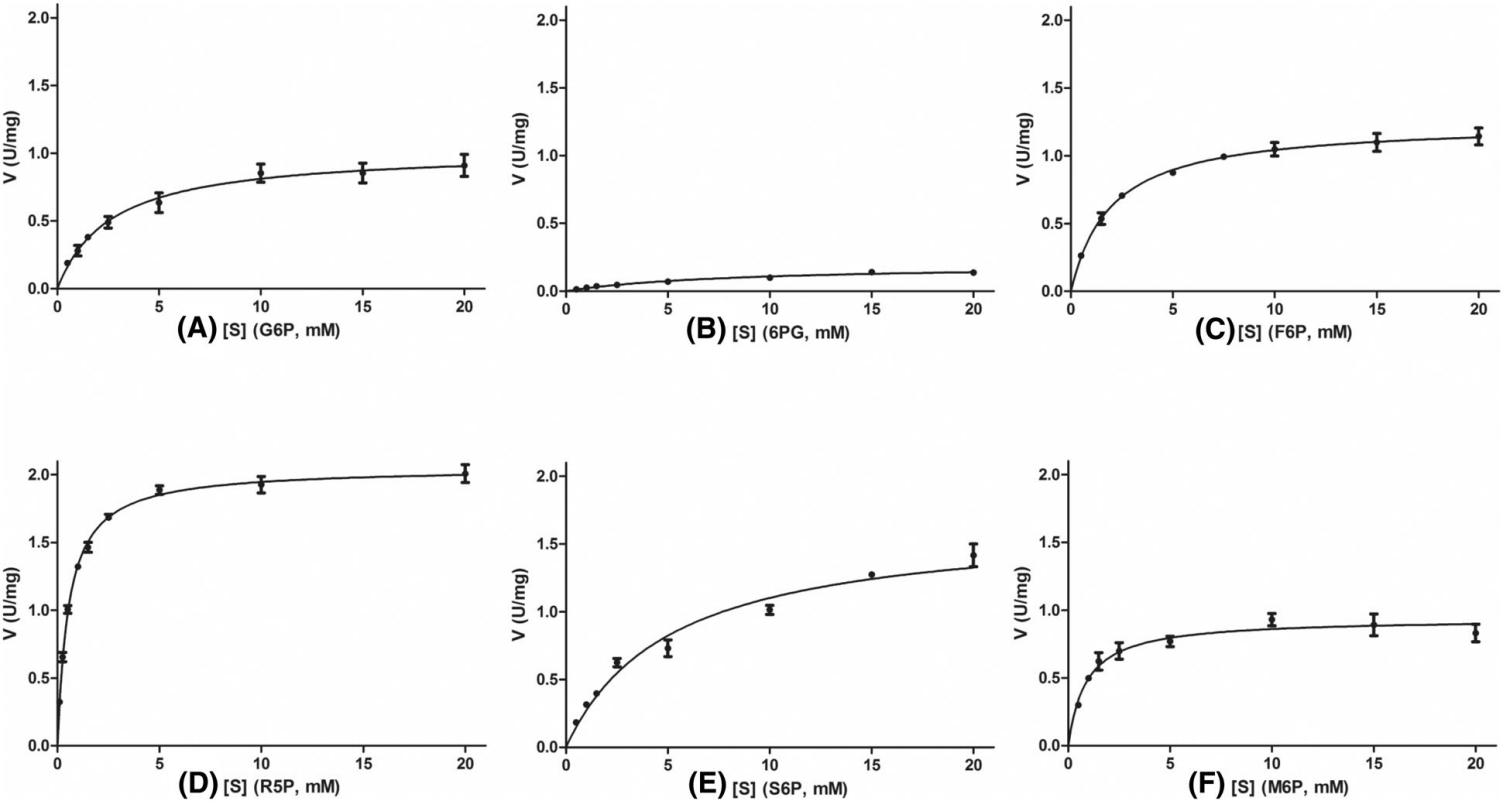
Substrate saturation curves of human recombinant G6PC3 using different sugar phosphates as substrates. *V* is the initial phosphatase reaction rate in U·mg^–1^ protein, [*S*] is the initial substrate concentration in mm. Each point is the mean ± SEM of three independent measurements (*n* = 3). (A) glucose‐6‐phosphate (G6P); (B) 6‐phospho‐gluconate (6PG); (C) fructose‐6‐phosphate (F6P); (D) ribose‐5‐phosphate (R5P); (E) sorbitol‐6‐phosphate (S6P); and (F) mannose‐6‐phosphate (M6P).

For the characterization of G6PC3, we used human recombinant protein overexpressed in HEK293T cells. To analyze the Michaelis–Menten kinetics of the enzyme, we plotted the substrate saturation curve of multiple sugar phosphates with non‐linear regression. The potential substrates we investigated, including their curve fit, were the following: glucose‐6‐phosphate (G6P, Sy.x = 0.086), ribose‐5‐phosphate (R5P, Sy.x = 0.06), 6‐phospho‐gluconate (6PG, Sy.x = 0.01), fructose‐6‐phosphate (F6P, Sy.x = 0.068), sorbitol‐6‐phosphate (S6P, Sy.x = 0.086) and mannose‐6‐phosphate (M6P, Sy.x = 0.081) (Fig. 1). All assays were linear, with respect to incubation time, and were performed using the same batch of sample. Regarding the non‐linear regression analysis, every phosphorylated carbohydrate we examined was confirmed to be a substrate for G6PC3 with differing affinity (Table 1). For the canonical G6P, we measured a *K*_m_ of ~2.3 mm at pH 6.5, which is comparable to data from previous studies [6, 9]. R5P showed the highest affinity with a *K*_m_ of ~0.5 mm and a *V*_max_ two times higher than that of G6P. M6P and F6P also showed higher affinity for the enzyme than G6P with *K*_m_ values around ~1 and ~2 mm, respectively, whereas the *V*_max_ was similar to that of G6P. S6P appears to be a weaker substrate with a *K*_m_ of ~5.1 mm and a *V*_max_ 1.6 times higher than that of G6P. 6PG in our experiments was also able to bind to G6PC3 but with a low affinity; the *K*_m_ was found to be ~8 mm and the *V*_max_ is five times lower than that of G6P. In light of this data, G6PC3 appears to be a non‐specific phosphohydrolase. Among the compounds we investigated R5P, M6P and F6P were better substrates for the enzyme than G6P, based on their catalytic efficiency (*V*_max_/*K*_m_) values, as shown in Table 1.

**Table 1:** G6PC3 Michaelis–Menten kinetics with catalytic efficiency (*V*_max_/*K*_m_). 6PG, 6‐phospho‐gluconate; F6P, fructose‐6‐phosphate; G6P, glucose‐6‐phosphate; M6P, mannose‐6‐phosphate; R5P, ribose‐5‐phosphate; S6P, sorbitol‐6‐phosphate.

Substrate	*K*_m_ (mm) (mean ± SE)	*V*_max_ (U·mg^–1^) (mean ± SE)	*V*_max_/*K*_m_ (10^−3^ min^–1^·mg^–1^)
G6P	2.32 ± 0.17	1.02 ± 0.05	0.44
6PG	8.22 ± 1.4	0.19 ± 0.01	0.02
F6P	1.96 ± 0.22	1.24 ± 0.03	0.64
R5P	0.54 ± 0.02	2.05 ± 0.02	3.78
S6P	5.11 ± 0.7	1.66 ± 0.08	0.33
M6P	0.89 ± 0.14	0.93 ± 0.03	1.05

### Transport measurements

**Fig. 2:**
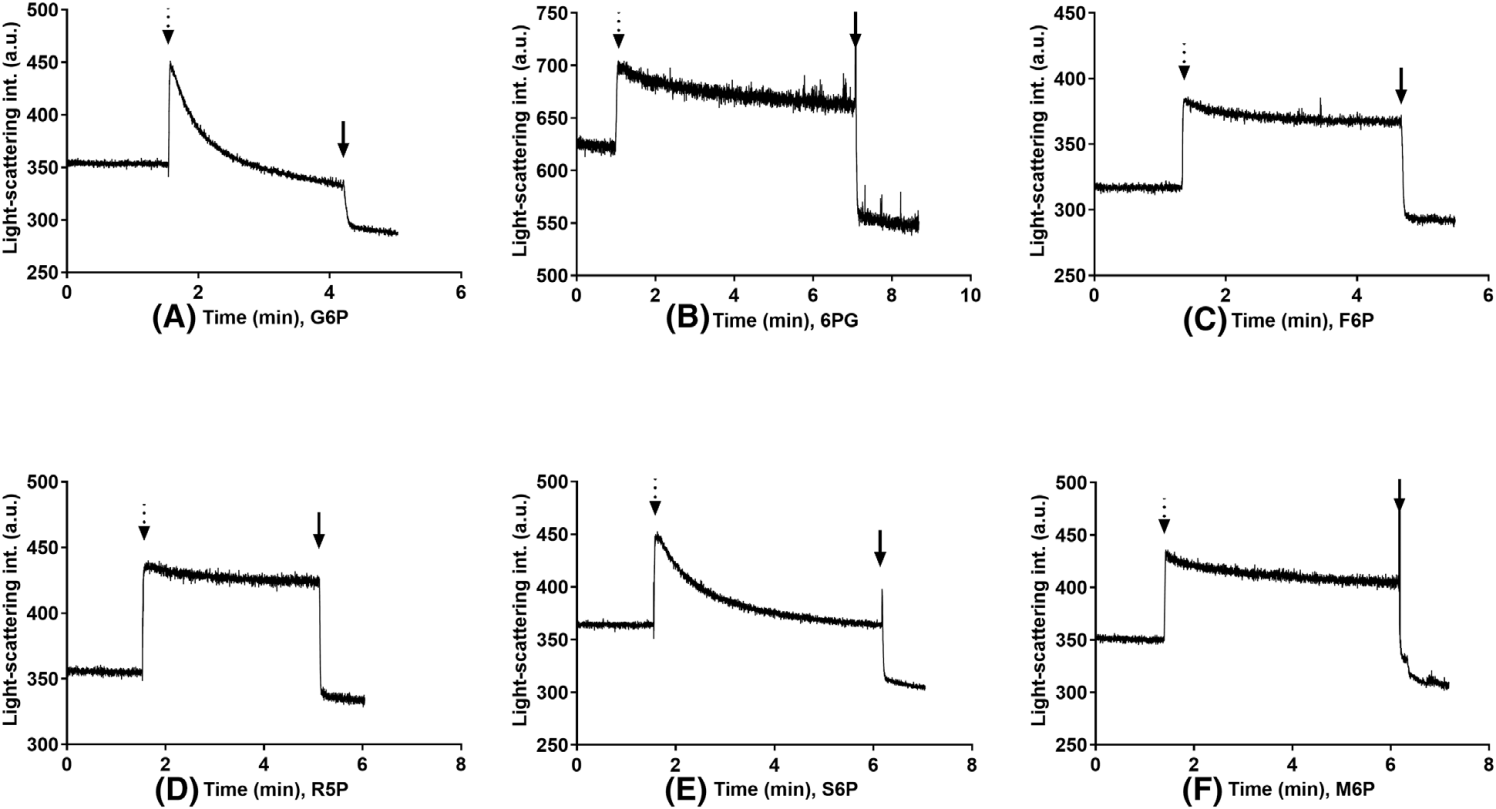
Changes in light‐scattering intensity of rat liver microsomes, osmotically induced by different sugar phosphates. The *y*‐axis shows light‐scattering intensity in an artificial unit and the *x*‐axis shows time elapsed. Concentrated solutions (1 m) of (A) glucose‐6‐phosphate (G6P); (B) 6‐phospho‐gluconate (6PG); (C) fructose‐6‐phosphate (F6P); (D) ribose‐5‐phosphate (R5P); (E) sorbitol‐6‐phosphate (S6P); and (F) mannose‐6‐phosphate (M6P) were added where indicated by the dotted arrows reaching a final concentration of 30 mm. At the end of the measurement, the microsomes were permeabilized by adding 2 μL of alamethicin (10 mg·mL^−1^) as indicated by the black arrows. A typical set of experiments out of three is shown (*n* = 3).

**Fig. 3:**
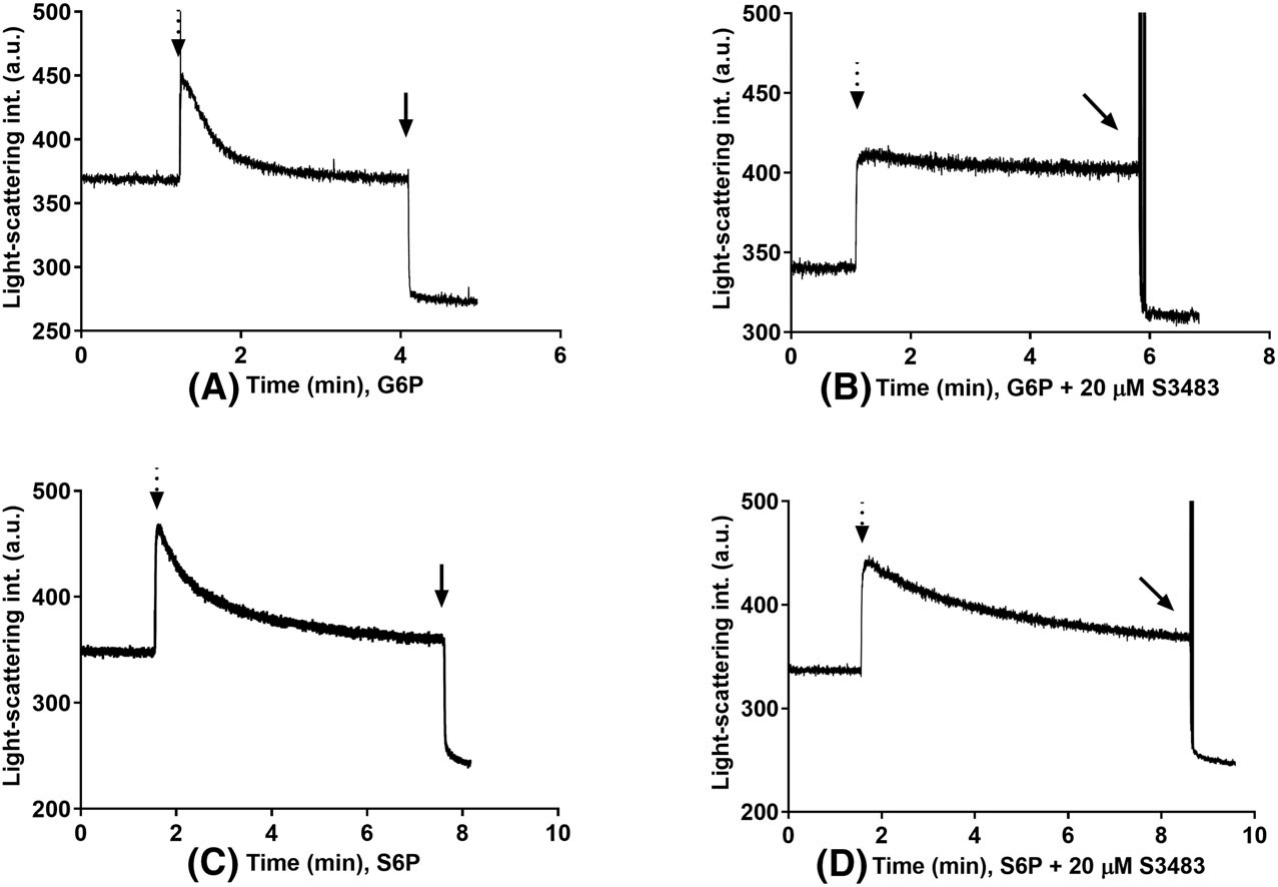
Effects of G6PT inhibitor S3483 on sorbitol‐6‐phosphate transport shown by osmotically induced changes in light‐scattering intensity of rat liver microsomes. The *y*‐axis shows light‐scattering intensity in an artificial unit and the *x*‐axis shows time elapsed. Concentrated solutions (1 m) of (A) glucose‐6‐phosphate; (B) glucose‐6‐phosphate with 20 μm S3483; (C) sorbitol‐6‐phosphate; and (D) sorbitol‐6‐phosphate with 20 μm S3483 were added where indicated by the dotted arrows. The final concentration was 30 mm. At the end of the measurement the microsomes were permeabilized by adding 2 μL of alamethicin (10 mg·mL^−1^) as indicated by the black arrows (*n* = 3).

For a substance to be a physiological substrate, we must consider its availability for the enzyme. Because G6PC3 has an active site facing the ER lumen, it works together with G6PT, which procures its substrates from the cytosol. To test the ER transmembrane transport of the compounds investigated, we conducted light‐scattering based transport measurements on rat liver microsome samples, which contain the ortholog of human G6PT as their main transport protein. Among the substrates we examined, G6P is known to be transported by G6PT [2], whereas M6P has low permeability; therefore, it is often used to evaluate microsomal intactness [25]. Regarding the other compounds, only S6P displayed transmembrane transport characteristics. F6P, R5P and 6PG showed low permeability, similar to M6P (Fig. 2), implying that the cytosolic supply of these metabolites is not transported efficiently by G6PT. Consequently, it cannot be hydrolyzed by G6PC3 in a significant manner. Interestingly, the transport of S6P was not blocked by S3483, a known specific G6PT inhibitor, as shown in Fig. 3. This suggests the presence of a protein not yet described in rat liver microsomes, which can transport S6P.

### Investigation of expression and activity changes of the G6PT‐G6PC3 system during neutrophil granulocyte differentiation

**Fig. 4:**
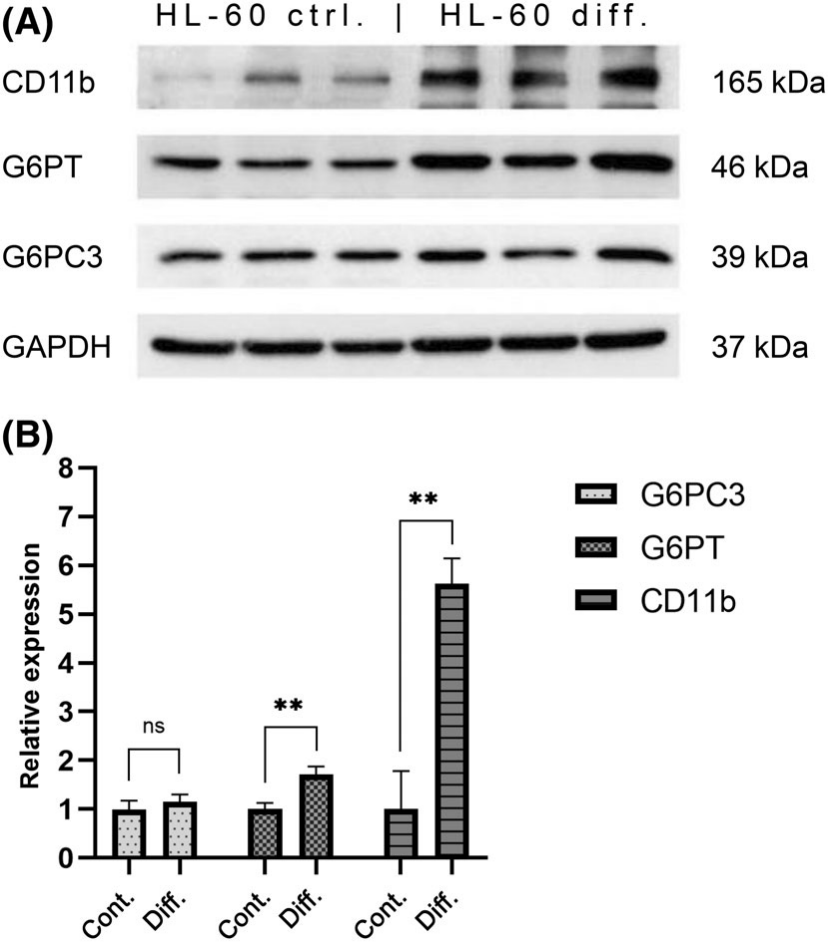
(A) Western blot analysis showing the protein expression of G6PT and G6PC3 in control (HL‐60 ctrl.) and differentiated (HL‐60 diff.) HL‐60 cells. The differentiation was carried out by treating cells with 1.25 vol% dimethylsulfoxide for 6 days. CD11b was used as an indicator of successful differentiation, GAPDH was the loading control. The bands shown are the result of three independent experiments (*n* = 3). (B) Relative protein expression difference between control and differentiated HL‐60 cells for G6PC3, G6PT and CD11b based on densitometric analysis of western blots shown in Fig. 4A. Data are the mean ± SEM of three sets of independent measurements. Comparisons were made using unpaired Student's *t*‐test. **P = 0.01–0.001. ns, non‐significant.

**Fig. 5:**
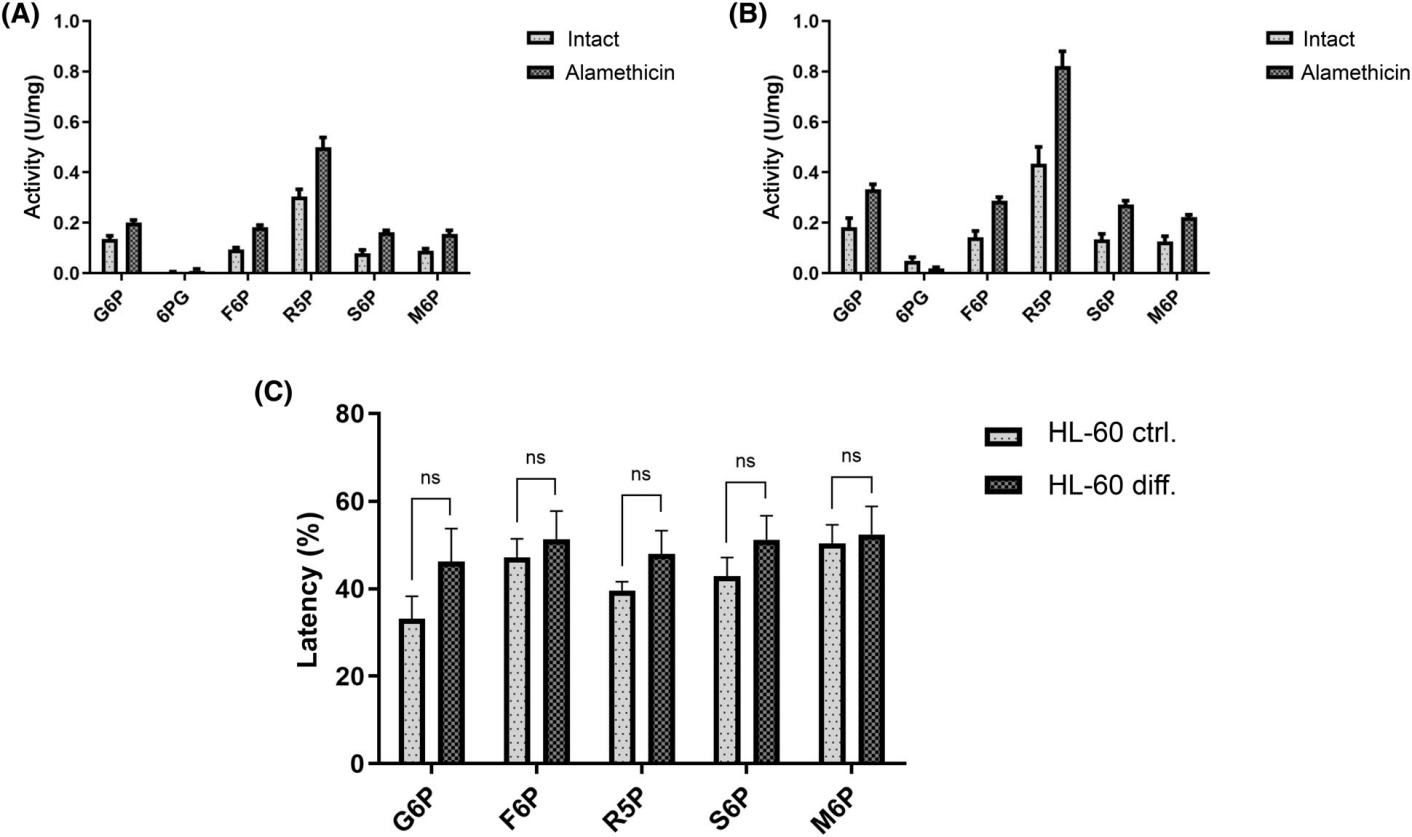
Phosphatase activity (U/mg protein) of intact and alamethicin (0.1 mg·mg^–1^ protein) permeabilized microsomes in (A) control and (B) differentiated HL‐60 cells using glucose‐6‐phosphate (G6P); 6‐phospho‐gluconate (6PG); fructose‐6‐phosphate (F6P); ribose‐5‐phosphate (R5P); sorbitol‐6‐phosphate and mannose‐6‐phosphate (M6P) as substrates. Data are the mean ± SEM of three sets of measurements (*n* = 3). (C) Phosphatase activity latency between control and differentiated HL‐60 cell derived microsomes using the substrates glucose‐6‐phosphate (G6P); fructose‐6‐phosphate (F6P); ribose‐5‐phosphate (R5P); sorbitol‐6‐phosphate and mannose‐6‐phosphate (M6P). Latency is calculated as: (activity_permeabilized_ − activity_intact_)/activity_intact_ × 100 (*n* = 3). Data are the mean ± SEM of three sets of measurements. Comparisons were made using unpaired Student's *t*‐test. ns, non‐significant.

To model neutrophil granulocyte differentiation, we used a HL‐60 cell line. HL‐60 cells are promyelocytes but can be differentiated into end stage neutrophil granulocyte‐like cells using 1.25 vol% dimethylsulfoxide. During this process, many morphological and functional changes occur, including increased CD11b integrin expression, which is a good marker of successful differentiation [26, 27, 28, 29]. We examined the changes in protein expression levels of G6PT and G6PC3 after a 6‐day differentiation of HL‐60 model cells with western blot analysis. According to our densitometric data, G6PT expression was increased by 1.7 ± 0.1235 (*P* = 0.0046), whereas the expression of G6PC3 did not change significantly (1.155 ± 0.133, *P* = 0.31) (Fig. 4). In addition, we investigated any potential changes in microsomal phosphatase activity latency between control and differentiated cells using HL‐60 derived microsomes. We found no significant changes in activity latency between the two groups (Table S1), as illustrated in Fig. 5. Despite the increment in the expression of G6PT, the ER intraluminal phosphatase activity stays constant during HL‐60 differentiation, supporting our hypothesis that the G6PT/G6PC3 system plays an equally important role from the promyelocytic stage onward.

## Discussion

In the present study, we aimed to investigate the substrate specificity of G6PC3 for different sugar phosphates, as well as the microsomal transport of these compounds. On the other hand, we clarified the protein expression changes of the G6PT‐G6PC3 system during neutrophil granulocyte differentiation using a human promyelocytic leukemia model (i.e. HL‐60) cell line.

Although 1,5‐AHG6P was shown to be an important physiological substrate for G6PC3 with a *K*_m_ of ~0.43 mm, the enzyme appears to be a non‐specific phosphohydrolase. Therefore, we performed kinetic studies using recombinant G6PC3 and found that, besides G6P, it was capable of dephosphorylating F6P, R5P, M6P, 6PG and S6P with different affinities. Our findings not only corroborate the kinetic data provided by Veiga et al. [9], but also complement them. At pH 6.5, we measured slightly higher, but comparable *K*_m_ values for G6P (*K*_m_ ~2.3 mm) and R5P (*K*_m_ ~0.5 mm) to the aforementioned study, affirming that R5P is a strong substrate for G6PC3. We also obtained new kinetic data demonstrating that F6P (*K*_m_ ~1 mm) and M6P (*K*_m_ ~2 mm) have higher affinity for G6PC3 than G6P. Although we measured phosphatase activity using S6P (*K*_m_ ~5,1 mm) and 6PG (*K*_m_ ~8 mm), based on their high K_m_ and relatively low catalytic efficiency values, it is unlikely that they are part of this alternative metabolic pathway.

The catalytic site of G6PC3 is facing the lumen of the ER; consequently, every potential substrate has to be either transported from the cytosol or generated intraluminally. G6PC3 works together with the ubiquitously expressed G6PT/SLC37A4, which transports its cytosolic substrates into the ER [2, 4, 5, 6]. To examine the ER transmembrane transport of the compounds investigated via G6PT, we conducted light‐scattering based transport measurements using rat liver derived microsomes. G6PT is also present in neutrophil granulocytes, exhibiting liver‐like transport kinetics [2, 8]. Among the substrates that we examined, F6P, R5P and 6PG showed low permeability because they were not transported rapidly by G6PT. S6P could enter the microsomes; moreover, this transport was not blocked by the inhibition of G6PT with its specific inhibitor S3483.

The F6P, R5P and 6PG intracellular metabolism mainly takes place in the cytosol. As metabolic intermediates, they are part of the cytosolic pentose phosphate pathway responsible for NADPH generation [30]. F6P was shown to be able to enter rat liver microsomes; however, the transport rate appeared to be approximately 10 times lower than that of G6P. In parallel, F6P was shown to induce NADPH generation in permeabilized rat liver microsomes, similarly to G6P [31]. In our transport measurements, F6P, R5P and 6PG did not show significant microsomal transport efficiency, suggesting that the G6PT‐G6PC3 system cannot dephosphorylate the cytosolic pool of these substances with great efficiency. Among these three compounds, the ER intraluminal generation of 6PG is well‐established because it can be created from G6P by hexose‐6‐phosphate dehydrogenase producing NADPH, which is partly used for glucocorticoid activation [32, 33]. The further metabolization of intraluminal 6PG is unclear. However, according to our kinetic data, there is convincing evidence that microsomal 6PG will not go through dephosphorylation by G6PC3. The ER intraluminal generation of F6P and R5P is possible because the enzymes of the pentose phosphate pathway were shown to be present in the ER; however, the activity of the cycle appeared to be only 1.5% of its cytoplasmic equivalent [34].

In our measurements, M6P appeared to be a better substrate for G6PC3 than G6P; however, it is not readily available for the enzyme. Liver microsomes are known to have poor permeability for M6P [25], which is supported by the results of our transport measurements. Additionally, there is no available data on intraluminal M6P generation in the ER.

Regarding S6P, it was a slightly weaker substrate for G6PC3 than G6P. In the cytoplasm, glucose can be reduced in the polyol pathway by aldose‐reductase, making sorbitol. Afterwards, sorbitol is mainly oxidized into fructose by sorbitol dehydrogenase. Additionally, sorbitol is also a substrate for hexokinase, making S6P generation possible [35, 36, 37]. Alternatively, G6P, as a substrate for aldose reductase, could also be converted directly into S6P [38, 39]. To date, there are no data available on S6P generation in the ER, but, according to our measurements, the cytosolic S6P appears to be able to enter rat liver microsomes, although at a lower rate than G6P. To conclude, S6P in our study was transported into the ER but, based on its high *K*_m_ (~5.1 mm) and relatively low catalytic efficiency value, the evidence suggests it does not go through dephosphorylation by G6PC3.

Recent research highlights the critical function of G6PC3 in neutrophil granulocytes by eliminating the toxic metabolite 1,5‐AHG6P. Inactivating mutations in G6PC3 lead to not only increased peripheral apoptosis and depressed effector functions, but also disturbed maturation of neutrophil granulocytes. This suggests that the G6PT‐G6PC3 system is indispensable at all stages of the neutrophil granulocyte life cycle [9, 13, 14]. Thus, we examined the changes in protein expression levels of G6PT and G6PC3 during the differentiation of HL‐60 model cells. After 6 days of differentiation, we found a ~1.7 fold increment in G6PT expression, whereas the expression of G6PC3 did not change significantly. In addition, we also assessed the potential changes in microsomal phosphatase activity latency between control and differentiated cells using HL‐60 derived microsomes. The increment in G6PT expression did not appear to influence the microsomal phosphatase activity, which is demonstrated by the lack of significant activity latency elevation. These data affirm the importance of a constant G6PC3 phosphatase activity in myeloid progenitor cells, as well as in peripheral neutrophil granulocytes.

The ubiquitously expressed G6PC3 enzyme is anchored in the ER by nine transmembrane domains, making protein purification challenging. Therefore, we used cell lysates for our measurements as described above, which makes all our *K*_m_ and *V*_max_ values apparent. On the other hand, we used rat liver derived microsomes to model the ER transmembrane transport catalyzed by G6PT/SLC37A4. Although the protein is highly conserved between species, rat derived microsomes might have slightly different transport characteristics from human derived ones. Finally, in the literature, HL‐60 cells are commonly used for modeling neutrophil granulocyte differentiation and function *in vitro*; however, it is important to consider that these cells do not offer a complete representation of *in vivo* neutrophil granulocytes. These limitations need to be considered in the interpretation of our results.

## Conclusions

This new kinetic study on G6PC3 using carbohydrate‐phosphates found that the enzyme is able to dephosphorylate other compounds than G6P *in vitro*. Our results are in agreement with recent findings, where G6PC3 is declared as a metabolic repair enzyme with a crucial function in neutrophil granulocytes eliminating 1,5‐AHG6P. Our observations do not rule out other possible physiological substrates for the enzyme. The significance of microsomal carbohydrate‐phosphate dephosphorylation needs to be further investigated in detail to determine whether G6PC3 is exclusively a metabolic repair enzyme or whether it takes part in other physiological metabolic pathways not yet described. Finally, we have demonstrated that G6PC3 expression and intraluminal phosphatase activity remain constant during HL‐60 differentiation despite the increment in G6PT expression. This corroborates the importance of a steady presence of G6PC3 activity throughout different maturation states. These results provide us with a clearer picture about the potential roles of the G6PT‐G6PC3 system and might open up new research directions in carbohydrate metabolism of neutrophil granulocytes.

## Conflicts of interest

The authors declare that they have no conflicts of interest.

### Peer review

The peer review history for this article is available at https://www.webofscience.com/api/gateway/wos/peer‐review/10.1002/2211‐5463.13924.

## Author contributions

ZsL conceived and designed the project, acquired, analyzed and interpreted the data, and wrote and edited the paper. KN edited the figuress. TK supervised the project.

## Supporting information


**Table S1.** Latency statistics.

## Data Availability

The data included in this study are available via: https://osf.io/645mb/?view_only=bd76ed3d5a43400ba5927ccf4321493f.
