# Supplementary material for: Biochemical characterization of the human ubiquitous glucose‐6‐phosphatase in neutrophil granulocytes
Source: FEBS Open Bio. 2024 Nov 15;15(2):285–95. doi: 10.1002/2211-5463.13924 (PMC11788743; doi:10.1002/2211-5463.13924)
Supplement: Supplementary file 1 — Table S1. Latency statistics. [file FEB4-15-285-s001.docx]

| **Table S1**  *Latency statistics* | | | | |
| --- | --- | --- | --- | --- |
| **Substrate** | **Microsome** | **Latency (%) mean ± SD** | **Confidence Interval**  **[lower – upper bound]** | ***p* value*** |
| **G6P** | Undiff. HL-60 | 33.16 ± 12.58 | 19.96 - 46.36 | 0.1728 |
|  | Diff. HL-60 | 46.26 ± 15.04 | 22.33 - 70.18 |  |
| **F6P** | Undiff. HL-60 | 47.15 ± 10.43 | 36.2 - 58.1 | 0.5834 |
|  | Diff. HL-60 | 51.34 ± 12.78 | 31 - 71.69 |  |
| **R5P** | Undiff. HL-60 | 39.65 ± 4.864 | 34.54 - 44.75 | 0.1306 |
|  | Diff. HL-60 | 47.94 ± 10.77 | 30.81 - 65.07 |  |
| **S6P** | Undiff. HL-60 | 42.88 ± 9.523 | 31.05 - 54.7 | 0.256 |
|  | Diff. HL-60 | 51.26 ± 10.83 | 34.02 - 68.5 |  |
| **M6P** | Undiff. HL-60 | 50.45 ± 9.281 | 38.93 - 61.98 | 0.7917 |
|  | Diff. HL-60 | 52.47 ± 12.79 | 32.12 - 72.81 |  |
| *Note*. * indicates unpaired *t*-test. SD = standard deviation; undiff. = undifferentiated; diff. = differentiated; G6P = glucose-6-phosphate; F6P = fructose-6-phosphate; R5P = ribose-5-phosphate; S6P = sorbitol-6-phosphate; M6P = mannose-6-phosphate. (n = 3) | | | | |
